# Intracranial Hematoma Detection by Near Infrared Spectroscopy in a Helicopter Emergency Medical Service: Practical Experience

**DOI:** 10.1155/2017/1846830

**Published:** 2017-06-22

**Authors:** Patrick Schober, Sebastiaan M. Bossers, Lothar A. Schwarte

**Affiliations:** ^1^Department of Anesthesiology, VU University Medical Center, De Boelelaan 1117, 1007 MB Amsterdam, Netherlands; ^2^Trauma Center and Helicopter Emergency Medical Service (HEMS) Lifeliner 1, VU University Medical Center, De Boelelaan 1117, 1007 MB Amsterdam, Netherlands

## Abstract

In (helicopter) emergency medical services, (H)EMS, the prehospital detection of intracranial hematomas should improve patient care and the triage to specialized neurosurgical hospitals. Recently, noninvasive detection of intracranial hematomas became possible by applying transcranial near infrared spectroscopy (NIRS). Herein, second-generation devices are currently available, for example, the Infrascanner 2000 (Infrascan), that appear suited also for prehospital (H)EMS applications. Since (H)EMS operations are time-critical, we studied the Infrascanner 2000 as a “first-time-right” monitor in healthy volunteers (*n* = 17, hospital employees, no neurologic history). Further, we studied the implementation of the Infrascanner 2000 in a European HEMS organization (Lifeliner 1, Amsterdam, The Netherlands). The principal results of our study were as follows: The screening for intracranial hematomas in healthy volunteers with first-time-right intention resulted in a marked rate of* virtual* hematomas (false positive results, i.e., 12/17), rendering more time consuming repeat measurements advisable. The results of the implementation of the Infrascanner in HEMS suggest that NIRS-based intracranial hematoma detection is feasible in the HEMS setting. However, some drawbacks exist and their possible solutions are discussed. Future studies will have to demonstrate how NIRS-based intracranial hematoma detection will improve prehospital decision making in (H)EMS and ultimately patient outcome.

## 1. Introduction

In emergency services, including helicopter emergency medical services (HEMS), the prehospital detection of intracranial hematomas could improve prehospital therapy and the triage of patients to specified neurosurgical hospitals [1, 2]. Recently, noninvasive detection of intracranial hemorrhage became possible by applying transcranial near infrared spectroscopy (NIRS) [3–8]. The first generation of these devices were hampered with several limitations [9] and a second generation is currently available, which by ruggedized design and advanced technology appears promising also for the prehospital setting. Only few studies are published on this technology to detect intracranial hematomas, particularly using the second-generation Infrascanner 2000 [10]. Herein the few studies available are mainly performed in hospital conditions [11, 12] and not in the prehospital setting of HEMS.

HEMS operations are utterly time-critical [13], for example, concept of the* Golden Hour*, and prolonged or repeated on-scene measurements should be avoided. Moreover, the head of trauma patients is routinely immobilized during transport, for example, with head blocks and head straps, limiting access to the head measurement points. We therefore studied the Infrascanner 2000 with the intention of “first-time-right” measurements. This is a modification of the practice suggested by the manufacturer to repeat every measurement up to 3 times, to rule out possible false positive measurements. So far, no publications are available hereon.

Together, the aims of this pilot study were (1) to study the use of the Infrascanner 2000 as a first-time-right monitor device and (2) to study the implementation of the Infrascanner 2000 in the Dutch HEMS setting.

## 2. Materials and Methods

### 2.1. Infrascanner

**Figure 1:**
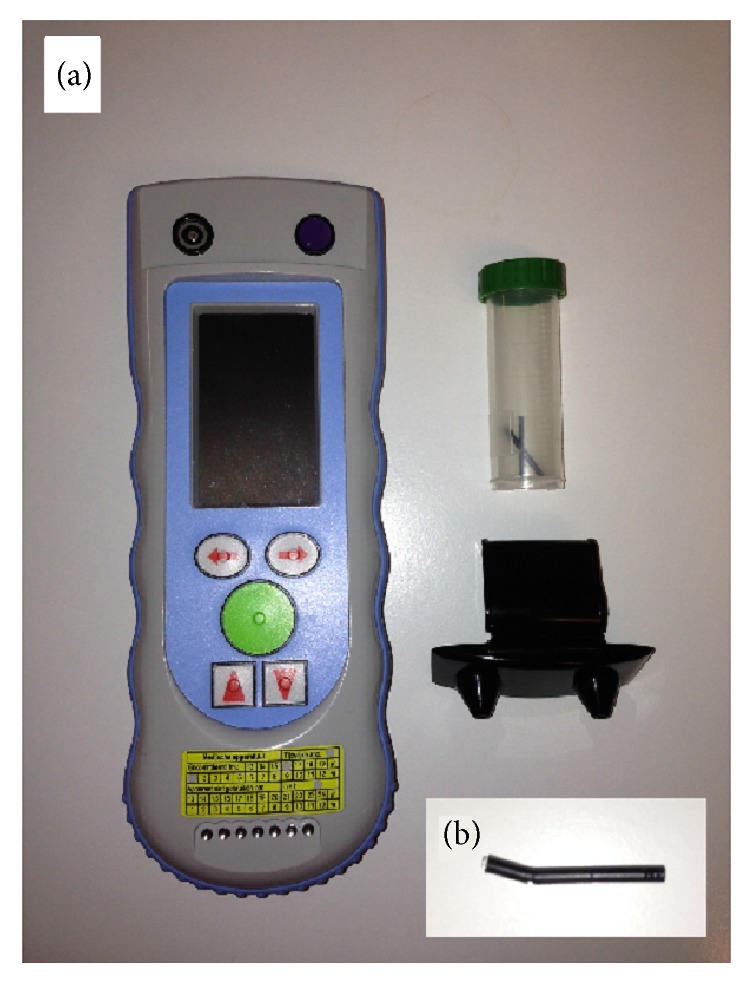
In (a), the Infrascanner 2000 (left side) is depicted with the disposable components, that is, click-on mount (right side, bottom) and the two optical fibers (right side top, in a transparent casing). In the HEMS setting, we currently transport the optical fibers in this casing to avoid snagging and breaking of mounted optical fibers. Inlay (b) shows such a broken light fiber.

All measurements to detect (intra)cranial hematomas were performed with the Infrascanner 2000 (Infrascan Inc., Philadelphia, USA) (Figure 1). According to manufacturer specifications, the Infrascanner 2000 detection limits are 3.5 mL for minimal volume, 2.5 cm for maximum depth from brain-surface, and 3.5 cm for maximum depth from skin-surface.

### 2.2. Volunteers

**Figure 2:**
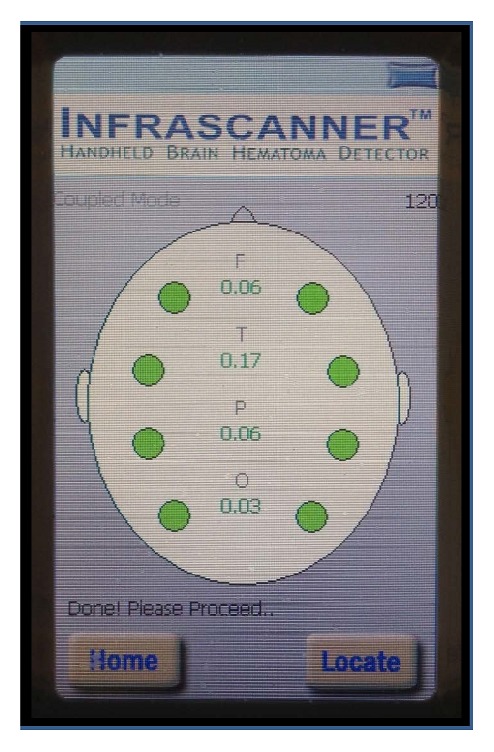
Example of an Infrascanner measurement in a healthy volunteer. The 4 bilateral measurement locations (letter code: F, frontal; T, temporal; P, parietal; O, occipital) results in 8 individual measurement spots. The green dots indicate that no hematoma is detected and the green numbers the neglectable optical left/right differences.

Infrascanner measurements were performed on *n* = 17 healthy volunteers, that is, adult hospital personnel. After obtaining informed consent, the Infrascanner measurements were performed in the individuals on 8 anatomical spots, yielding 4 measurement pairs per volunteer (Figure 2):

- Frontal spot: above the centered pupil, below the hair line
- Temporal spot: temporal fossa, just anterior to the top of the ear
- Parietal spot: midway between top of the ear and top of the skull
- Occipital spot: midway between top of the ear and the occipital protuberance.

The detection algorithm of the Infrascanner for intracranial hematomas includes the assumption that strictly symmetrical anatomical measurement points (i.e., on the left and right side) yield a comparable light absorption. An unilaterally increased light absorption might be caused by an intracranial hematoma. However, to minimize the chance that minor asymmetries either in the measurement spot location or in cranial anatomy will result in falsely detected hematomas, the manufacturer preprogrammed a cut-off for optical side differences (delta 0.2) into the system.

### 2.3. HEMS Field Study

The Infrascanner was temporarily implemented (January–August 2016) in the regional, physician staffed HEMS organization (Lifeliner 1, University Medical Center Amsterdam, The Netherlands). This HEMS operation serves the entire Northwest region of the Netherlands of about 4.5 million inhabitants. In 2016, this HEMS operation was dispatched 3337 times. The major HEMS indications were traffic accidents (46%) and high energy falls (26%), both prone to traumatic brain injury.

For safety reasons, all new electrical devices used in HEMS services are tested prior to implementation for possible interference with existing on-board medical equipment and avionics of the helicopter (Eurocopter EC-135, Airbus industries) both on ground and in flight [14].

After safety testing, the Infrascanner was implemented into the HEMS operation as follows: Upon a dispatch, the Infrascanner was placed in the HEMS physician's uniform pocket and carried to the patients. Selection of the individual HEMS physician to apply the Infrascanner was based on individual judgement per patient.

## 3. Results

### 3.1. Volunteers

Infrascanner measurements with the first-time-right intention were performed in *n* = 17 individual volunteers, all adult employees of the hospital. In 5 of 17 volunteers, the initial measurement confirmed absence of intracranial hematomas (exemplified in Figure 2). However, in 12 of 17 volunteers, the initial measurement suggested an intracranial hematoma, mostly at the 3 posterior measurement spots. The false hematomas detected in the volunteers were generally close to the preprogrammed detection cut-off value of 0.2.

### 3.2. HEMS Field Study

**Figure 3:**
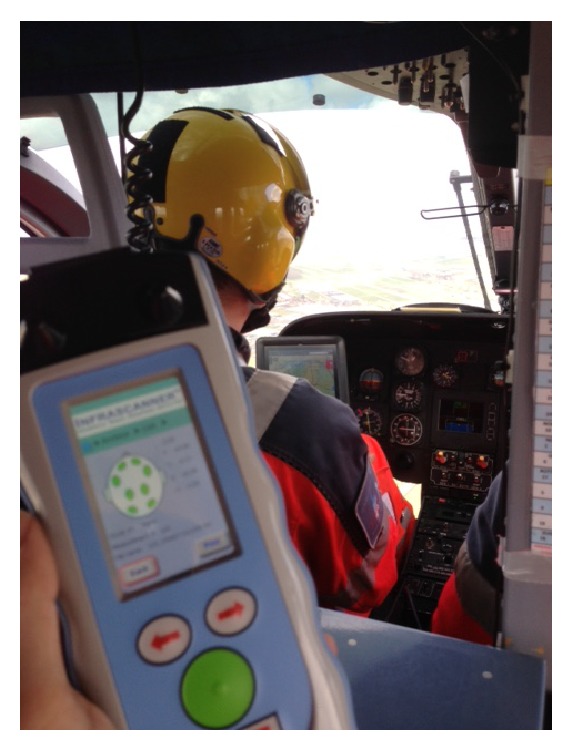
The Infrascanner 2000 was implemented in a Dutch helicopter emergency medical service (HEMS). For safety reasons, the device was tested prior to implementation for possible interference with existing on-board medical equipment, electronics, and avionics of the helicopter (Eurocopter EC-135, Airbus industries) both on ground and during flight.

The results regarding flight safety testing were as follows: Testing for possible interferences of the Infrascanner 2000 with existing on-board medical equipment, board electronics, and avionics did not show any abnormalities both on the ground and during flights. In turn, there were no disturbances of the Infrascanner recorded caused by the HEMS environment on ground and during flight (Figure 3).

### 3.3. Device Handling

**Figure 4:**
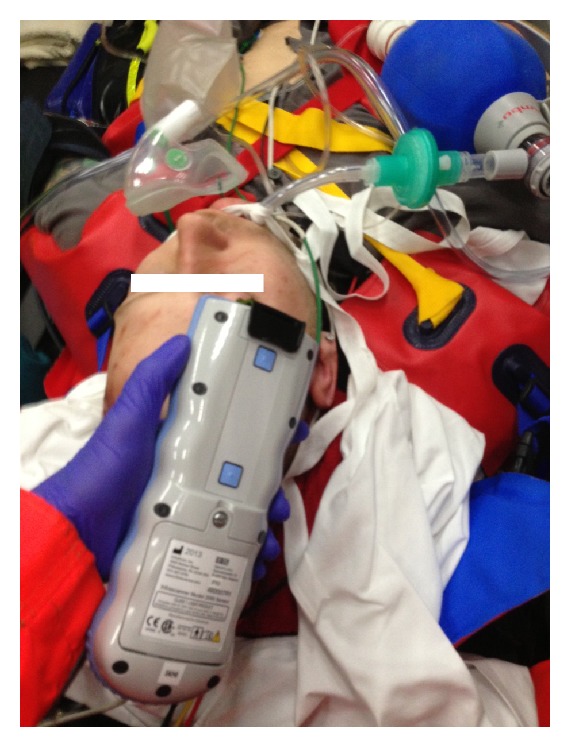
Example of an Infrascanner measurement of a sedated, intubated motor-cross rider in the HEMS setting. Here, the right frontal (i.e., supraorbital) spot is accessed for the measurement. Placing the patient on a vacuum matrass for spine immobilization maintained accessibility to the head for Infrascanner measurements.

The HEMS physicians reported a sufficient ease to handle the system, regarding both on-scene assembly and ergonomics of use, for example, regarding weight and dimensions (Figures 3 and 4). In detail, however the users also reported minor handling issues with the disposable click-on shield: The optical fibers stick out of the shield, allowing the fibers to get snagged, bent, or break (Figure 1(b)). Additionally the fibers are somewhat mobile in the cap for adjusting the optical system, occasionally allowing the fibers to dislocate from the shield.

### 3.4. HEMS Use of the Infrascanner in Trauma Patients

**Figure 5:**
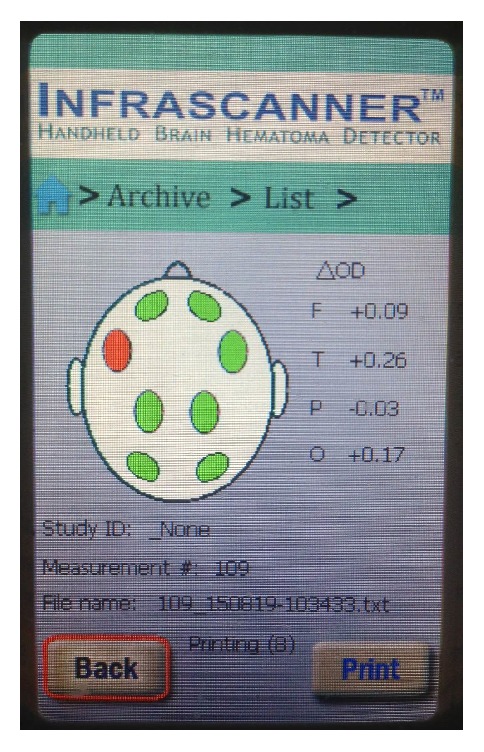
Example of an Infrascanner measurement of a sedated, intubated motor-cross rider in the HEMS setting. This prehospital measurement suggested an intracranial hematoma in the left parietal region (red dot), which was later confirmed by the in-hospital CT scan.

**Figure 6:**
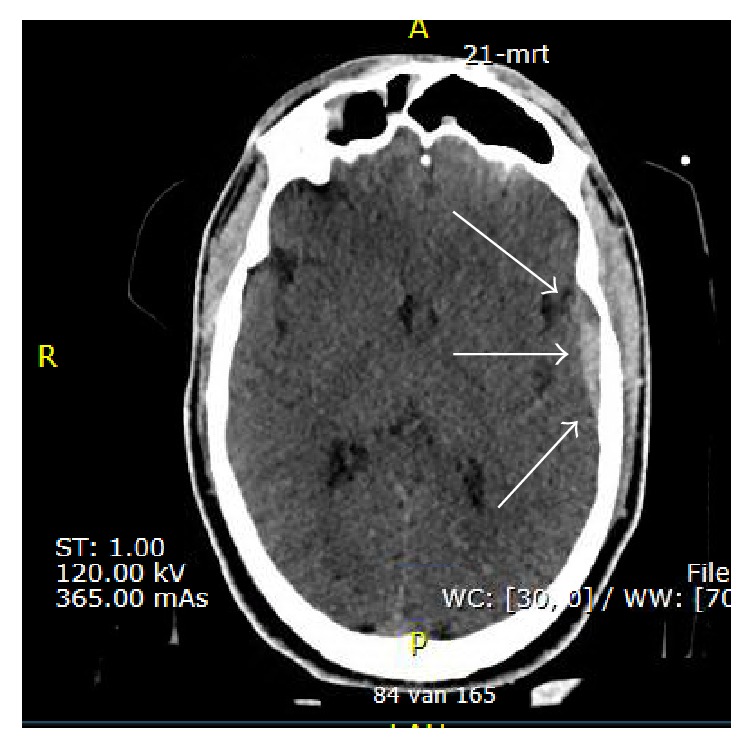
Cranial CT scan of a motor-cross rider, in which the prehospital Infrascanner measurement suggested an intracranial hematoma in the left parietal region. The cranial CT confirms this suspicion (white arrows). Cave: by convention, the CT image is left/right flipped, in respect to the Infrascanner image.

**Figure 7:**
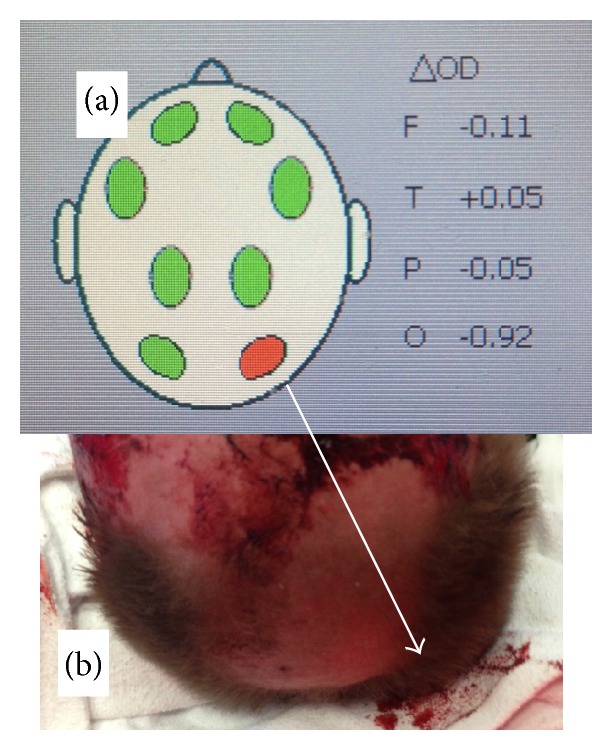
Photography (b) of a traffic victim's head (truck driver). Indicated (by white arrow) is a lacerated, occipital hematoma (as seen from cranial direction). This subcutaneous hematoma was part of the Infrascanner catchment volume, leading to the suggestion of an intracranial hematoma at this same spot ((a), Infrascanner screenshot). An intracranial hematoma was not confirmed by in-hospital cranial CT, stressing the need to meticulously circumvent subcutaneous hematomas at the measurement sites.

The exact number of patients assessed with the Infrascanner during this pilot implementation remained unrecorded, since the measurements have not always been documented in the HEMS organization's database. Therefore, we present qualitative rather than quantitative description, with results compiled from HEMS physician's reports. The HEMS physicians reported numerous cases in which prehospital Infrascanner measurements suggested cranial hematomas, which were later confirmed by in-hospital CT scans (e.g., Figures 4–6). However, also sub- and supragaleal hematomas were classified as intracranial hematomas, when Infrascanner measurements were performed in that area (Figure 7).

**Figure 8:**
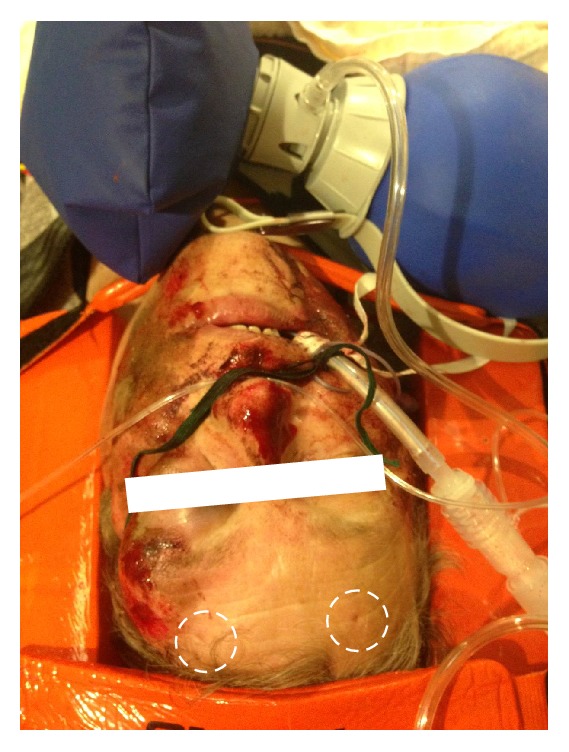
Photography of an intubated, ventilated trauma patient (scooter driver) with suspected neurotrauma. The traditional cervical spine immobilization with head blocks and head straps hinders access to the standard Infrascanner measurement spots, except for the supraorbital region. In addition, the left supraorbital region is affected by an orbital hematoma (the CT confirmed an orbital fracture), making the Infrascanner measurements challenging. The dashed circles mark pin-point impressions that originate from the Infrascanner light fiber tips.

**Figure 9:**
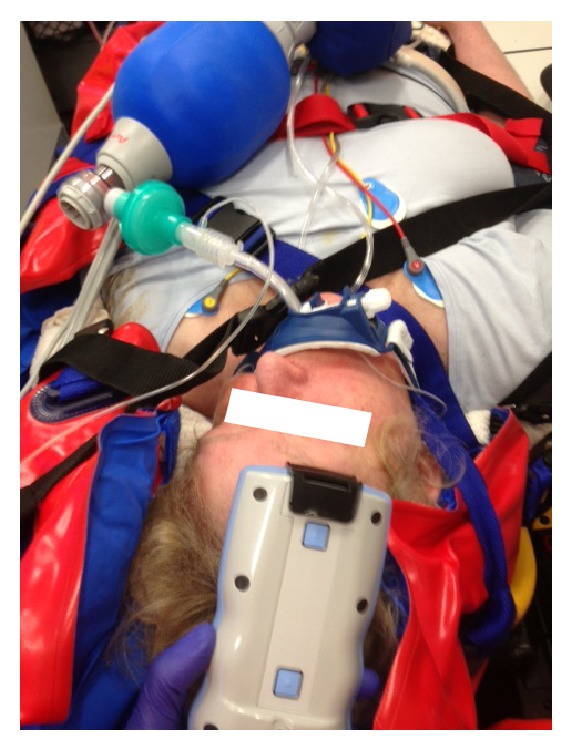
Photography of an intubated, ventilated trauma patient with suspected neurotrauma during an Infrascanner examination. The alternative cervical spine immobilization with a vacuum matrass allows an improved access to all Infrascanner measurement points, except for the most occipital measurement points.

In patients requiring traditional cervical spine fixation with head blocks and head straps on a spine board, access to the head measurement points suggested by the manufacturer is hindered, except for the frontal, supraorbital measurement point (Figure 8). Particularly repeat measurements were undesired since they delayed spine immobilization or patient transport and were thus regularly omitted. For the same reason of hindered accessibility, follow-up measurements during the patient transport to the hospital were not performed. However, the access to the Infrascanner measurement points was greatly improved, when the trauma patients were immobilized on vacuum matrasses, complying to updated trauma protocols (Figure 9).

### 3.5. Infrascanner Measurements in Awake Children and Handicapped Patients

In *n* = 3 cases, the HEMS physicians attempted to perform measurements in awake children and mentally handicapped patients after a suspected neurotrauma. In these trauma cases, the patients became agitated and uncooperative, when the Infrascanner's optical fibers were firmly applied onto their skin. No complete measurement set could be performed in these 3 patients.

## 4. Discussion

The application of NIRS-based technologies is currently used to assess tissue oxygenation [15] and increasingly, as presented here, to screen for intracranial hematomas. Screening for intracranial hematomas in the HEMS setting should improve prehospital patient treatment and triage to specialized neurosurgical hospitals.

In the HEMS setting, we tested the NIRS-based Infrascanner 2000 and found it a (flight-) safe, portable (e.g., lightweight and size), and technically feasible technique for implementation in HEMS organizations. However, based on the results of this pilot study, several suggestions were derived, as discussed below.

The results from this pilot study indicate that the Infrascanner 2000 is not yet suitable as a first-time-right monitor; for example, there is a high percentage of cases, where the initial measurement suggested intracranial hematomas in healthy volunteers (false positive, virtual hematomas). In a screening tool, a relatively high rate of false positive results may be acceptable if the aim is to reliably identify all (or most) patients with a pathological condition, as here intracranial hematomas. However, our initial measurements resulted in 70% virtual hematomas in healthy volunteers, a high percentage even for screening methods. Although we cannot exclude that additional training will reduce the frequency of initial false positive measurements, it appears that technical modifications are required to allow that already the first measurement obtains correct results. Thus, at this point, the users should follow the manufacturer's advice to perform control measurements to cross check initial positive findings.

The virtual hematomas in healthy volunteers were generally close to the preprogrammed detection cut-off of 0.2, indicating that minor software adjustments could eliminate a major portion of these false positive results. This would increase the specificity, but care must be taken that the detection sensitivity remains in clinical acceptable range.

In addition, these virtual hematomas predominantly occurred in the 3 rearward measurement spots, compared to the frontal spot, suggesting the following explanations: Hair is absent on the frontal measurement spot but covers the 3 rearward measurement spots in most cases. If hair sticks between skin and the Infrascanner's light fiber, light absorption will be disturbed, contributing to the virtual hematomas. Therefore, removing hair at the measurement spots should reduce this source of artifacts.

Finally, the anatomic definition of the frontal measurement point is rather exact and reproducible compared to the three rearward measurement points. This supports symmetric measurements on the frontal spot and more asymmetric measurements on the posterior measurement spots. Since left/right asymmetries in the catchment volume contribute to left/right absorption differences, this also contributes to the calculation of virtual hematomas.

In head trauma patients, the Infrascanner suggested (intra)cranial hematomas, in cases when only subcutaneous scalp hematomas were present. This phenomenon has been described before, and it is recommended to measure outside these subcutaneous hematomas. However, this attempt poses practical challenges: First, the edge of a subcutaneous hematoma is frequently not visible from outside; thus defining safe measurement distances from the hematoma's edge can be challenging. Secondly, the location of a subcutaneous hematoma regularly corresponds to the site of traumatic impact. Thus, an intracranial hematoma might be obscured behind the superficial subcutaneous hematoma and thus diagnostically missed.

In children in the hospital, measurements with the Infrascanner have been described before [12, 16, 17]. However, the measurements with the Infrascanner might take several minutes [16] and involve the unpleasant, firm application of the light fiber tips on the patient's scalp. In our few HEMS cases, we failed to obtain complete measurements, likely because the trauma setting did not allow the patients to sufficiently calm down. Children and (other) uncooperative patients should be instructed and calmed before the procedure, when possible. Although seldom applicable in the HEMS setting, skin preparation with a local anesthetic crème, for example, EMLA® or Rapidan®, might increase patient comfort during the Infrascanner measurements.

The traditional cervical spine immobilization by tightly fixating the patient's head with head blocks and head straps to a spine board hindered the measurement access with the Infrascanner, except for the frontal (supraorbital) measurement spot. However, based on recent (inter)national trauma protocol modifications, trauma patients are increasingly immobilized by vacuum matrasses. In the future, this will allow a better access to the trauma patient's head, allowing better option for Infrascanner measurements, except for the most occipital measurement spots. That way, Infrascanner measurements become also possible in the immobilized patient during transport to the hospital.

## 5. Conclusion

In conclusion, our pilot study shows that prehospital NIRS-based intracranial hematoma detection is a promising technique, feasible to be implemented in a HEMS organization. With our practical modifications described, the studied Infrascanner 2000 system is portable, rugged, and sufficiently ergonomic for application in the prehospital setting.

However, our study also demonstrates current drawbacks of the system for the (H)EMS setting. Our data in healthy volunteers show a marked percentage of virtual hematomas when we tested the system as a “first-time-right” monitor. Thus, this attempt to minimize the time required for the hematoma screening proved unsuccessful and more time consuming (repeat) measurements have to be taken in account. Technical advances and other developments may improve this aspect in the future.

Furthermore, the Infrascanner proved sensitive for subcutaneous and subgaleal hematomas. Thus care must be taken that those blood deposits remain outside the NIRS-assessed volume. In trauma patients, however, relevant intracranial hematomas may remain obscured and thus undetected behind superficial hematomas. In addition, our study addresses potential Infrascanner limitations in other special patient categories, that is, in pediatric trauma patients and patients with chronic intracranial hematomas.

Future studies will have to demonstrate how prehospital NIRS-based intracranial hematoma detection can support HEMS physician's decisions, for example, regarding prehospital treatment algorithms and triage, to ultimately improve patient outcome.
